# *Vernonia amygdalina* as a hop substitute in red sorghum beer: Effects on fermentation performance and physicochemical properties

**DOI:** 10.1016/j.crfs.2026.101331

**Published:** 2026-02-02

**Authors:** Arthur K. Amisi, Nathan Luyeye, Jean-Paul Koto-Te-Nyiwa Ngbolua, Exaucé Matundu, Guelor Kasereka, Jean-Claude T. Bwanganga

**Affiliations:** a Fermentation and Distillation Laboratory, Department of Chemistry and Agricultural Industries, Faculty of Agricultural Sciences and Environment, University of Kinshasa, PO Box. 14071, Kinshasa 1, Democratic Republic of the Congo; b Research-Innovations Laboratory in Agri-Food Industries (LRIIAA), Bandundu-Ville, Democratic Republic of the Congo; c Department of Biology, Faculty of Science and Technology, University of Kinshasa, Kinshasa, Democratic Republic of the Congo; d Department of Agri-Food Sciences, Faculty of Agronomy and Veterinary Medicine, Loyola University of Congo, Democratic Republic of the Congo; e Faculty of Agronomy, Forestry and Environmental Sciences, Bel Campus Technological University, 8 ème Rue Limete Industriel, PO Box. 94, Limete, Kinshasa, Democratic Republic of the Congo

**Keywords:** Hops, ***Vernonia amygdalina***, Beer, Fermentation, Brewing, Sorghum beer, Bitterness

## Abstract

The substitution of imported hops with locally available botanical alternatives offers a sustainable strategy for sorghum-based beer production. This study evaluated partial and total replacement of hops with *Vernonia amygdalina* as a bittering agent on fermentation performance and physicochemical properties of red sorghum malt beer. Five formulations were prepared with 0, 25, 50, 75, and 100% (w/w) substitution and fermented for 192 h using *Saccharomyces cerevisiae* (brewer's yeast strain ANGEL BF-16) under controlled laboratory conditions.

*V. amygdalina* was added at the end of the 60-min boil, immediately before cooling, to maximize extraction of bitter compounds while preserving heat-sensitive aromatic molecules. Fermentation kinetics and final beer characteristics were assessed via extract content, density, alcohol concentration, pH, color (EBC), and apparent attenuation. Increasing proportions of *V. amygdalina* significantly enhanced fermentation, yielding higher alcohol content (5.2–6.3% v/v), increased apparent attenuation (61.6–75.5%), and lower final pH values compared to the hop-only control. Higher substitution levels also increased color intensity, reflecting contributions of plant-derived pigments and polyphenols.

Microbiological analyses confirmed the absence of detectable contaminants, indicating that *V. amygdalina* did not inhibit yeast activity. Statistical analysis showed that fermentation duration and bitter extract formulation significantly affected all measured variables (p < 0.05). Although sensory evaluation and detailed phytochemical profiling were not performed, results indicate that *V. amygdalina* is a viable hop substitute with measurable effects on fermentation efficiency and beer quality. Future studies should investigate sensory acceptance and optimize substitution levels for industrial application.

## Highlights

• Hop substitution with a local natural resource (*Vernonia amygdalina*)
• Alcohol yield improved by 21% thanks to the use of *V. amygdalina*
• Improved apparent attenuation and alcohol production were observed with increasing *V. amygdalina* substitution.

## Graphical abstract

**Figure:**
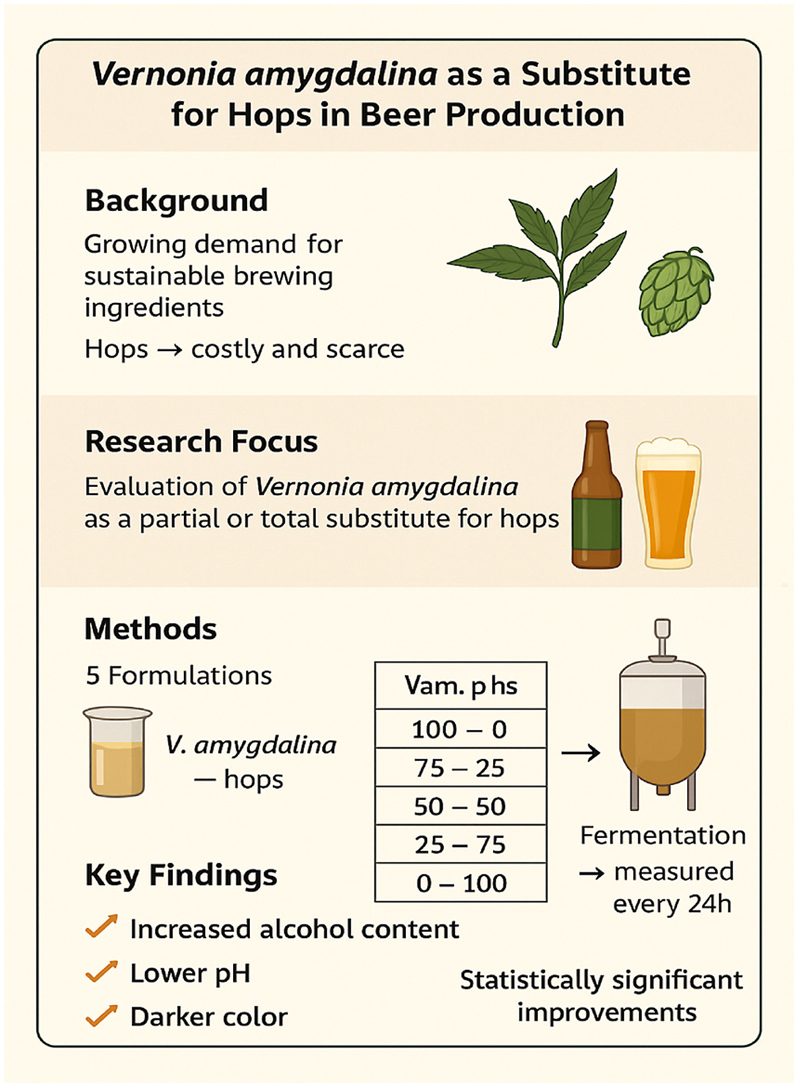

## 1 Introduction

Beer production relies traditionally on hops (*Humulus lupulus* L.) as a key ingredient providing bitterness, aroma, microbiological stability, and foam retention (Amisi et al., 2023). However, hop cultivation is geographically restricted and highly dependent on specific climatic conditions, making many brewing regions—particularly in sub-Saharan Africa—structurally dependent on imported hop products (Amisi et al., 2024). This dependency increases production costs and limits the development of locally adapted brewing systems. Consequently, there is growing interest in identifying alternative bittering agents derived from locally available plant resources that could partially or totally replace hops while maintaining acceptable fermentation performance and product quality (Desobgo et al., 2013; Shishir and Chen, 2017; Sapara et al., 2022).

The variability of hop bitterness and polyphenolic profiles is strongly influenced by cultivar, growing region, and terroir (Herkenhoff et al., 2024a; He et al., 2014). These factors lead to measurable differences in beer color, acidity, aroma, and fermentation behavior, highlighting the concept of terroir in brewing. From this perspective, the use of *Vernonia amygdalina* should not be viewed solely as a direct “replacement” for hops, but rather as an alternative botanical bittering source whose effects may be conceptually comparable to the variations observed when changing hop origin or chemical composition (Van Holle et al., 2021).

The variability of hop bitterness and polyphenolic profiles is strongly influenced by cultivar, growing region, and terroir. Recent HS-SPME/GC-MS analyses have confirmed that hop terroir plays a decisive role in shaping aroma composition and technological behavior in beer, resulting in distinct volatile profiles and fermentation responses depending on geographical origin (Herkenhoff et al., 2024b). These findings highlight that botanical origin is a critical driver of brewing performance and underscore the relevance of exploring region-specific bittering substitutes adapted to local raw materials and production systems.

In parallel, sorghum (*Sorghum bicolor* L. Moench) has emerged as a strategic cereal for brewing in tropical regions due to its drought tolerance, agronomic resilience, and suitability for gluten-free beer production (Chantereau et al., 2013; Hossain et al., 2022). Sorghum malt has been widely studied as a base material for both industrial and traditional beers, yet its technological limitations—particularly lower enzymatic activity and variable fermentability—often require process optimization or ingredient supplementation (Amisi et al., 2023, 2024). Within this context, the choice of bittering agents may play a dual role by contributing not only to sensory attributes but also to fermentation kinetics and microbial stability (Bwanganga, 2013; Cheriet and Mouhoub, 2012).

Among potential botanical alternatives, *Vernonia amygdalina* Delile, commonly known as bitter leaf, is a perennial plant widely distributed in Africa and traditionally used for its bitter taste, medicinal properties, and antimicrobial activity (Farombi and Owoeye, 2011; Amisi et al., 2023). Phytochemical analyses have shown that *V. amygdalina* leaves contain sesquiterpene lactones, polyphenols, flavonoids, and mineral compounds, many of which are known to influence microbial growth and metabolic activity (Bayoï and Etoa, 2023; Cadenas et al., 2021). These properties suggest that *V. amygdalina* could function as a multifunctional brewing adjunct, providing bitterness while potentially modulating yeast performance and microbial ecology during fermentation (Amisi et al., 2023; Ruangchawalee, 2018).

Previous studies have explored the use of *V. amygdalina* in traditional opaque beers or as an additive for antimicrobial purposes (Bayoï et al., 2022; Okafor and Aniche, 1983; Okoro, 1993). However, most of these works focused either on qualitative sensory observations or on post-fermentation additions, without systematically evaluating fermentation kinetics, alcohol production, and physicochemical evolution under controlled brewing conditions. The successful development of tropical hop substitutes from mixtures of bitter leaves, including *V. amygdalina*, has been reported, demonstrating acceptable beer quality in terms of bitterness, aroma, and microbial stability (Okoro, 1993; Amisi et al., 2023). Furthermore, the addition of *V. amygdalina* extracts has been shown to influence alcohol content and yeast growth positively (Amisi et al., 2023) while also enhancing sensory characteristics and extending shelf life in traditional sorghum beers (Bayoï et al., 2022).

*V. amygdalina*'s bioactive compounds, such as sesquiterpenes and lactones, impart pronounced bitterness, which can significantly alter the sensory profile of beer. Moderate concentrations may enhance aromatic complexity, whereas excessive levels could lead to unpleasant bitterness (Lyumugabe et al., 2012). Moreover, extracts of *V. amygdalina* exhibit antimicrobial activity against bacteria and fungi without negatively affecting brewer's yeast, supporting its potential role in improving microbial stability (Babalola and Okoh, 1996; Farombi and Owoeye, 2011).

Despite the growing interest in botanical alternatives to hops, quantitative data on the progressive substitution of hops with *Vernonia amygdalina* in clarified sorghum beer brewing remain scarce. Most previous studies have focused on traditional opaque beers, post-fermentation additions, or qualitative sensory observations, with limited investigation of fermentation kinetics, alcohol yield, and physicochemical evolution under controlled brewing conditions. Moreover, the potential dual role of *V. amygdalina*—as a bittering agent and as a source of bioactive compounds capable of modulating yeast metabolism and microbial stability—has not been systematically assessed in sorghum malt wort.

Therefore, the present study aimed to quantify the effect of partial and total substitution of hops with *Vernonia amygdalina* on fermentation performance and physicochemical properties of red sorghum beer. Specifically, the objectives were to:

(i) evaluate the influence of increasing proportions of *V. amygdalina* on fermentation kinetics, apparent attenuation, alcohol production, pH evolution, and color development;
(ii) assess the impact of hop substitution on microbiological quality at the end of fermentation under controlled laboratory conditions.

Based on the known phytochemical composition of *V. amygdalina* and its reported antimicrobial and nutritional properties, we hypothesized that:

(H1) increasing substitution levels of hops with *V. amygdalina* would enhance apparent attenuation and alcohol yield without inhibiting *Saccharomyces cerevisiae* activity; and

(H2) *V. amygdalina* would contribute to microbiological stability by limiting contaminating microorganisms while maintaining fermentation performance within acceptable brewing ranges.

This study is positioned as an exploratory technological assessment of gradual hop substitution, without implying chemical or sensory equivalence with conventional hops, and aims to contribute to current discussions on local bioresources, ingredient origin, and sustainable brewing systems in sorghum-based beer production.

## 2 Materials and methods

### 2.1 Materials

The sorghum used in this study is an ecotype of red sorghum (*Sorghum bicolor* L. Moench) sold locally in the city of Goma in North Kivu and intended for traditional brewing. Grains with insect damage or other defects, as well as harvest residues, were removed by visual inspection. The grains had a density of 1.1886, water sensitivity of 99%, water absorption of 48.0%, germination energy of 100, germination index of 7.9, and germination capacity of 100%.

The yeast used for fermentation was *Saccharomyces cerevisiae* ANGEL BF-16 (Angel Yeast Co., Ltd., China), 24 h old. The inoculum was standardized to an optical density at 600 nm (OD_600_) of 0.01, corresponding to approximately 0.005 g/L of dry yeast biomass, based on OD–biomass conversion. The strain was provided by the Center for Research and Innovation Specializing in Agri-Food and Cosmetics Industries (CRISIAC), University of Kinshasa.

The hops used in this study were pelletized hops (pellets) from an ecotype of *Humulus lupulus* (Saaz variety, noble hops, α-acid content ≈ 4%), supplied by Charles Faram & Co. Ltd. (Czech Republic) and purchased in Belgium for use at CRISIAC. The pelletized form was selected due to its homogeneous composition, ease of dosing, and reproducibility, which are particularly suitable for laboratory-scale brewing experiments.

*Vernonia amygdalina* leaves were obtained locally in Kinshasa, specifically at the large market of the National Pedagogical University (UPN), where various bitter essences are sold.

### 2.2 Methods

All brewing and fermentation experiments were conducted in triplicate as independent biological replicates. Each analytical measurement was performed on three independently fermented samples, and results are reported as mean ± standard deviation.

#### 2.2.1 Preparation of *Vernonia amygdalina* extract

The fresh leaves of *Vernonia amygdalina* were thoroughly washed with distilled water to remove impurities and surface residues, then left to drain at room temperature for 8 h. They were then sliced into small pieces and dried in an oven set at 40 °C for 72 h until they reached a stable weight, in order to preserve heat-sensitive compounds. The dried leaves were ground into powder using an electric mill, and the powder produced was then sieved to 0.5 mm.

#### 2.2.2 Extraction of phenolic compounds

Pending the development of physical methods for removing phenolic compounds—known to inhibit amylase activity in sorghum—a treatment based on a mixture of acetone/water solvents (70/30, v/v) was applied, in accordance with the method described by Bwanganga et al. (2012) and Amisi et al. (2023, 2024), with some modifications.

Thus, 2 kg of red sorghum grains were soaked in 2 L of solvent for 20 min, centrifuged at 5000 rpm for 5 min (Funke Gerber centrifuge), and rinsed with distilled water until the effluent was clear.

#### 2.2.3 Maltage and brewing

Rinsed sorghum grains (2 kg) were soaked in distilled water for 45 h using five cycles of 7-h soaking + 2-h airing. Grains germinated away from light, kilned at 40 °C for 48 h, then ground and sieved (1 mm).

After the malting process, the malt contained: α-amylase 255 U/g, β-amylase 50 U/g, β-glucanase 6 U/g, total phenolic 2.7 GAE/g, condensed tannins 0.07%, soluble nitrogen 42% (32% after 2 h boiling) (Amisi and Bwanganga, 2025).

Brewing was performed with 1650 g malt in 10 L distilled water (pH 5.6), adjusting wort density to 15 °P (1.0625). The 10 L wort was split into five 2 L containers and subjected to mashing: 62 °C/20 min (β-amylase), 72 °C/20 min (α-amylase), 100 °C/60 min boiling.

#### 2.2.4 Addition of bittering agents

The total amount of bittering material added was 7 g of dried powdered *V. amygdalina* were used per 2 L of wort, following a mass-based substitution approach, calculated based on hop usage to achieve a target bitterness of approximately 35 International Bitterness Units (IBU), corresponding to a typical Pilsner-style beer. Bitterness calculations were performed exclusively for hops, as IBU values are not applicable to *Vernonia amygdalina*; in this context, IBU served solely as a reference point. The hop variety used was Saaz (noble hops), with an alpha-acid content of approximately 4%. For a wort volume of 2 L and a boiling time of 60 min, the required hop extract mass was calculated considering alpha-acid isomerization yields of approximately 15% after 30 min and 25% after 60 min, according to the following equation:

(1) $$\text{Quantity}\;\text{Bitter}\;\text{Extract}\;\left(g\right)=\frac{35\;x\;2}{10\;x\;0.25\;x\;4}$$

This calculation resulted in a required bittering mass of 7 g per 2 L of wort.

In the absence of standardized bitterness units or characterized iso-alpha acid equivalents for *Vernonia amygdalina*, a mass-based substitution strategy was adopted. The total mass of bittering material was kept constant at 7 g per 2 L of wort, with partial or total replacement of hops by *V. amygdalina*. This approach was intentionally implemented as a technological screening strategy, assuming exploratory equivalence rather than chemical or sensory equivalence, to enable assessment of fermentation behavior and physicochemical responses to progressive hop replacement under controlled laboratory conditions. Five hop/*V. amygdalina* ratios (100–0, 75–25, 50–50, 25–75, and 0–100, w/w) were tested (see Supplementary Table 1).

The addition of *V. amygdalina* was performed after the boil, during wort cooling and just before fermentation. This timing was selected to preserve its delicate bitter compounds while avoiding the development of overly strong vegetal or astringent flavors, ensuring a balanced bitterness and maintaining the aromatic integrity of the final product.

The experiment followed a two-factor factorial design comprising five bittering ratios and nine fermentation time points, with three biological replicates per condition, resulting in a total of 135 experimental units.

#### 2.2.5 Fermentation

After boiling, the worts were cooled to 20 °C and inoculated with *Saccharomyces cerevisiae* ANGEL BF-16 at a deliberately low pitching rate (OD_600_ = 0.01), corresponding to approximately 0.005 g L^−1^ of dry yeast biomass. This constrained inoculation level was intentionally applied to enhance the sensitivity of the fermentation system to differences in wort composition induced by partial or total substitution of hops with *Vernonia amygdalina*. Such an approach, commonly adopted in laboratory-scale comparative fermentations, enables the detection of subtle stimulatory or inhibitory effects on yeast metabolism under controlled laboratory conditions, rather than aiming to replicate industrial pitching practices.

Fermentation was conducted for 192 h at 20–30 °C in a Minifor laboratory fermentor of 2.5 L of capacity under continuous stirring at 150 rpm to ensure homogeneity, reproducibility, and consistent yeast–substrate contact at laboratory scale. Although stirred fermentation does not fully replicate industrial static brewing conditions, it is well suited for controlled laboratory-scale experiments. It should be emphasized that the use of mild agitation does not aim to mimic industrial brewing practice, but rather to ensure homogeneity and reproducibility under laboratory conditions; therefore, absolute fermentation kinetics should not be directly extrapolated to industrial-scale static fermentations. The fermentation temperature range (20–30 °C) was selected to reflect realistic ambient conditions commonly used in artisanal sorghum beer production in tropical regions, including the Democratic Republic of Congo, and to remain within the physiological tolerance range of the *Saccharomyces cerevisiae* strain employed, rather than strictly controlled industrial lager fermentation conditions.

During fermentation, sugar consumption, pH, density expressed as degrees Plato (°P), color, and alcohol content were monitored every 24 h. Density measurements expressed in °Plato were used consistently as the reference indicator of extract consumption throughout the study. Alcohol content was calculated from °Plato values according to Amisi and Bwanganga (2025).

(2) Density(°P)=[(d−1)×258.5]/[0.12+(0.88×d)]

Where d is density.

Real extract (RE, °P) was calculated using the Balling equation:

(3) RE(°P)=0.1808×Pe+0.8192×Pb

where **Pe** is the original extract (°Plato) and **Pb** is the final extract (°Plato).

Alcohol content was calculated as follows:

(4) Alcohol(%m/v,g/100mL)=(Pe−Pb)×0.794

Alcohol (% v/v, mL/100 mL) was calculated from alcohol (% m/v) using the standard density conversion factor.

(5) %alcohol(v/v:ml/100ml)=Alcohol(%m/v,g/100mL)/0.789

Apparent attenuation (%) was calculated as:

(6) Apparentattenuation(%)=[(Pe−Pb)/Pe]×100

#### 2.2.6 pH measurement and color determination

The hydrogen potential (pH), which reflects the acidity or alkalinity of a solution, is directly related to the concentration of H_3_O^+^ ions. In this study, the pH of the beer samples was measured using a Hanna pH meter (Edge model) following standard procedures.

The color of the beer, expressed in EBC units, was determined by UV–visible spectrophotometry at 430 nm in accordance with standard methods. Prior to measurement, the spectrophotometer was calibrated using a cuvette filled with distilled water. Each sample cuvette was rinsed with the beer sample, filled, and measured with distilled water as a blank. Beer color was then calculated according to the following equation:

(7) Color(EBC)=OD430x25

Where OD is optical density.

#### 2.2.7 Statistical analysis

The collected data were analyzed using Minitab 17 and Python software. A two-factor analysis of variance (ANOVA) was performed to evaluate the effects of bitter extract dose and fermentation time on all response variables, including pH, °Plato, color, density, actual extract, attenuation, and alcohol content.

The experimental design included nine levels of fermentation time (0, 24, 48, 72, 96, 120, 144, 168, and 192 h) and five levels of bitter extract ratio (100–0, 75–25, 50–50, 25–75, and 0–100 for hops and *V. amygdalina,* respectively). All experiments were conducted in triplicate, and results are reported as mean ± standard deviation.

Post-hoc comparisons were carried out using Tukey's HSD test at a 5% significance level (p < 0.05). Prior to ANOVA, data were checked for normality and homogeneity of variances to ensure validity of the analysis.

#### 2.2.8 Microbiological analysis

**Microbiological analyses** were performed as quality control tests to assess the presence of potential contaminating microorganisms in the finished beers at the end of fermentation (192 h). The objective was not to characterize the fermentation microbiota in detail, but to ensure compliance with basic microbiological safety criteria for fermented beverages.

Beer samples were collected aseptically immediately after completion of alcoholic fermentation, before any stabilization treatment. Approximately 150 mL of each homogenized sample was analyzed using membrane filtration through sterile 0.45 μm cellulose acetate membranes. The membranes were then transferred onto selective culture media for the enumeration of target microorganisms.

Total mesophilic aerobic bacteria were enumerated on Plate Count Agar (PCA) after incubation at 30 °C for 48 h, while yeasts and molds were enumerated on Sabouraud Dextrose Agar to detect potential non-*Saccharomyces* contaminants. The inoculated *Saccharomyces cerevisiae* strain was not targeted, as yeast growth and fermentation performance were monitored indirectly through fermentation kinetics, sugar consumption, and ethanol production. Thus, analyses focused on confirming the absence of undesirable microbial contaminants rather than on total yeast counts.

In addition, qualitative detection tests were conducted for specific contaminating microorganisms of technological and hygienic relevance in brewing, including coliform bacteria, sulfite-reducing clostridia, and lactic acid bacteria, using standard selective media and incubation conditions. For clarity, fermentation performance was assessed using independent physicochemical parameters, including sugar consumption, apparent attenuation, and ethanol production; therefore, the absence of total yeast cell counts does not affect the interpretation of the results. Results were reported as presence or absence per 150 mL of beer, corresponding to the detection limits of the applied methods.

It should be noted that these microbiological analyses focused exclusively on potential contaminants relevant to product safety and quality, and were not intended to quantify the inoculated *Saccharomyces cerevisiae* population. Yeast growth and activity were instead indirectly assessed through fermentation kinetics, sugar consumption, and ethanol production.

## 3 Results et discussions

### 3.1 Results

#### 3.1.1 Physicochemical analyses

The initial physicochemical parameters of the worts and the final characteristics of the beers produced with different hop/*Vernonia amygdalina* substitution ratios are summarized in Table 1. Treatments E1 to E5 correspond to increasing proportions of *V. amygdalina* as bittering agent, ranging from 0% (E1, 100% hops) to 100% (E5, no hops).

**Table 1:** Evolution of various parameters during fermentation.

	pH_0_	pH_f_	Initial Color (EBC)	Final color (EBC)	d_0_ (°P)	d_f_ (°P)	R.E (°P)	Attenuation (%)	%Alcohol (v/v)
E1	5.5 ± 0.01	4.39 ± 0.01^a^	9.7 ± 0.01^a^	8.0 ± 0.12^a^	15 ± 0.1	5.9 ± 0.04^a^	7.6 ± 0.04^a^	61.6 ± 0.3^a^	5.2 ± 0.02^a^
E2	5.5 ± 0.01	4.36 ± 0.04^ab^	10.2 ± 0.01^b^	8.3 ± 0.1^b^	15 ± 0.1	5.8 ± 0.22^a^	7.5 ± 0.2^a^	62.4 ± 1.5^a^	5.3 ± 0.11^a^
E3	5.5 ± 0.01	4.29 ± 0.01^b^	10.7 ± 0.01^c^	8.9 ± 0.01^c^	15 ± 0.1	4.4 ± 0.10^b^	6.4 ± 0.04^b^	71.5 ± 0.3^b^	6.0 ± 0.03^b^
E4	5.5 ± 0.01	4.24 ± 0.01^c^	11.2 ± 0.01^d^	9.5 ± 0.01^d^	15 ± 0.1	4.0 ± 0.13^c^	6.0 ± 0.10^c^	74.1 ± 0.8^c^	6.2 ± 0.06^c^
E5	5.5 ± 0.01	4.19 ± 0.01^d^	11.7 ± 0.01^e^	9.9 ± 0.01^e^	15 ± 0.1	3.8 ± 0.29^c^	5.8 ± 0.24^c^	75.5 ± 1.9^c^	6.3 ± 0.15^c^

Values are expressed as mean ± standard deviation (n = 3). Values sharing the same superscript letter within a column are not significantly different according to Tukey's HSD test (p < 0.05).

Where E1 is sample with 100% hops, E2 is sample with 75% hops and 25% *V.A*; E3 is sample with 50% hops and 50% *V.A*; E4 is sample with 25% hops and 75% *V.A*; E5 is sample with 100% *V.A*, pH0 is initial pH, pHf is final pH, d0 (°P) is initial density in degrees Plato; df (°P) is final density in degrees Plato, RE (°P) is real extract in degrees Plato.

Initial wort extract values were comparable across all formulations, indicating a consistent mashing and wort preparation process. Density evolution during fermentation was monitored exclusively using hydrometric measurements expressed in degrees Plato (°P), which were applied consistently throughout the study as the reference indicator of extract consumption and fermentation progress.

Initial pH values were similar among all treatments, suggesting that bittering agent substitution did not significantly affect wort pH prior to fermentation. This indicates that the observed differences in final acidity primarily resulted from fermentation activity rather than initial wort composition.

At the end of fermentation, beers produced with higher proportions of *V. amygdalina* exhibited significantly lower final pH values, higher alcohol content, higher apparent attenuation, and lower residual extract compared to the hop-only control (E1). The formulation containing 100% *V. amygdalina* (E5) showed the highest alcohol content (6.3% v/v), while the lowest value (5.2% v/v) was recorded in E1.

Color intensity (EBC) increased progressively with increasing levels of *V. amygdalina*, with the highest values observed in treatments E4 and E5. This trend reflects the contribution of plant-derived pigments and polyphenolic compounds from *V. amygdalina*.

#### 3.1.2 Fermentation kinetics

**Fig. 1:**
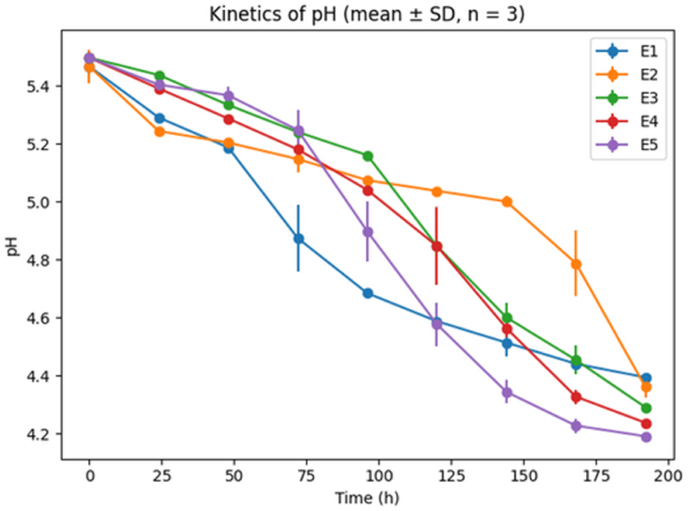
Kinetics of pH during fermentation Where E1 is sample with 100% hops, E2 is sample with 75% hops and 25% *V.A,* E3 is Sample with 50% hops and 50% *V.A*, E4 is Sample with 25% hops and 75% *V.A*, E5 is sample with 100% *V.A*. Time-course evolution of pH during fermentation for the different treatments (E1–E5). Values are expressed as mean ± standard deviation (SD) from three independent replicates (n = 3). A progressive decrease in pH was observed over time for all treatments, with variations in acidification rates depending on the experimental conditions.

**Fig. 2:**
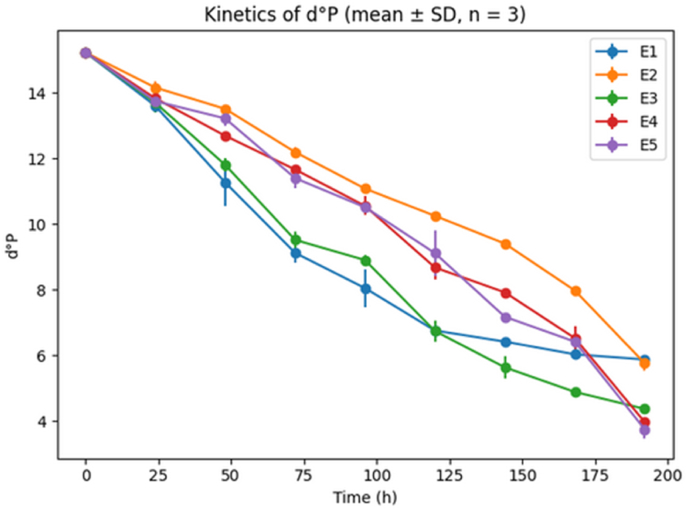
Kinetics of wort density (d°P) Where E1 is sample with 100% hops, E2 is sample with 75% hops and 25% *V.A,* E3 is Sample with 50% hops and 50% *V.A*, E4 is Sample with 25% hops and 75% *V.A*, E5 is sample with 100% *V.A*. Changes in wort density (degrees Plato, d°P), derived from hydrometric measurements, as a function of fermentation time for treatments E1–E5. Data represent mean ± SD (n = 3). A continuous decrease in d°P indicates the consumption of fermentable sugars, with distinct fermentation kinetics among treatments.

**Fig. 3:**
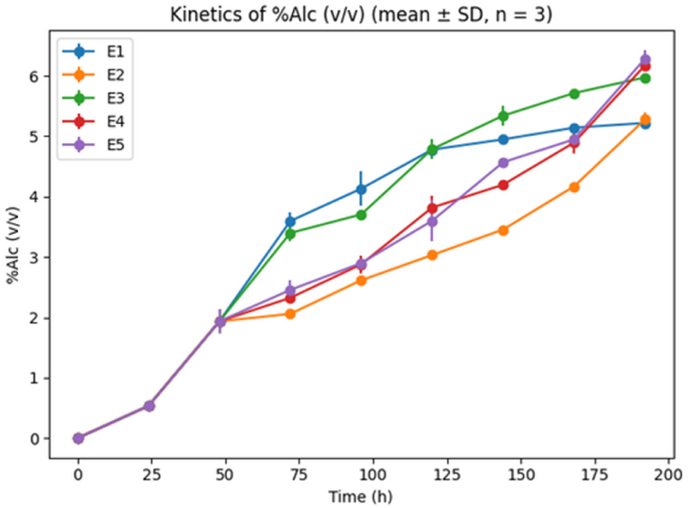
**Kinetics of alcohol production** Where E1 is sample with 100% hops, E2 is sample with 75% hops and 25% *V.A,* E3 is Sample with 50% hops and 50% *V.A*, E4 is Sample with 25% hops and 75% *V.A*, E5 is sample with 100% *V.A*. *Alcohol production kinetics (% v/v) during fermentation under different treatments (E1–E5). Results are presented as mean ± SD (n = 3). Alcohol concentration increased steadily throughout fermentation, reflecting yeast metabolic activity, with treatment-dependent differences in ethanol yield.*

Changes in extract content, density, pH, and alcohol concentration during fermentation are presented in Fig. 1, Fig. 2, Fig. 3. All results are expressed as mean values of three independent fermentations.

The pH decreased throughout fermentation for all treatments, reflecting yeast metabolic activity and organic acid production. Beers brewed with higher proportions of *V. amygdalina* reached lower final pH values, indicating increased acidification during fermentation.

Fig. 2 shows the evolution of density, expressed in degrees Plato (°P), for each sample (E1 to E5) as a function of fermentation time.

In all treatments, extract content decreased steadily throughout fermentation, indicating effective sugar consumption by *Saccharomyces cerevisiae*. However, formulations containing higher proportions of *V. amygdalina* showed a steeper decline in extract during the early and mid-fermentation phases, suggesting accelerated fermentation kinetics.

Density evolution followed a similar pattern, with a more rapid decrease observed in treatments E3–E5 compared to E1 and E2. These differences are consistent with the higher final attenuation and alcohol content measured in *V. amygdalina*-rich formulations.

Fig. 3 shows the evolution of alcohol content (% v/v) for samples E1 and E5 during fermentation.

Alcohol concentration increased progressively in all samples, with significantly higher final values in treatments containing larger amounts of *V. amygdalina*. This confirms that the substitution level influenced fermentation efficiency rather than merely altering final beer composition.

#### 3.1.3 Statistical analyses

Analysis of variance (ANOVA) revealed that fermentation duration and bitter extract dosage (hop/*Vernonia amygdalina* substitution level) had statistically significant effects (p < 0.05) on all measured response variables, including extract content, density, alcohol concentration, pH, color, and apparent attenuation. Significant interaction effects between fermentation time and bitter extract formulation were also observed, indicating that the influence of the bittering agent composition evolved throughout the fermentation process.

These results demonstrate that both bittering formulation and fermentation duration play critical roles in shaping the physicochemical characteristics of sorghum-based beer.

#### 3.1.4 Microbiological analyses

Microbiological quality control analyses performed at the end of fermentation (192 h) showed no detectable contaminating microorganisms in any of the beer samples (Table 2). Total mesophilic aerobic bacteria and non-*Saccharomyces* yeasts and molds were below the detection limits of the applied methods, while coliform bacteria, lactic acid bacteria, and sulfite-reducing clostridia were absent in all treatments.

**Table 2:** Results of microbiological analyses.

Target microorganisms	Result	Expression
Total mesophilic aerobic bacteria	Not detected	< detection limit/150 mL
Yeasts and molds (excluding *S. cerevisiae*)	Not detected	< detection limit/150 mL
Coliform bacteria	Absent	Absence/150 mL
Lactic acid bacteria	Absent	Absence/150 mL
Sulfite-reducing clostridia (pH 5 and 7)	Absent	Absence/150 mL

Detection limits correspond to the sensitivity of the membrane filtration method used. Results reflect quality control assessments of contaminating microorganisms and do not indicate sterility.

These results indicate that the fermentation process was conducted under hygienic laboratory conditions and that the presence of *Vernonia amygdalina* did not compromise microbiological safety. Alcohol production, attenuation, and density decrease confirmed the active presence and metabolic performance of the inoculated *Saccharomyces cerevisiae*, although yeast cell counts were not determined directly.

### 3.2 Discussions

The present study demonstrates that the progressive substitution of hops with *Vernonia amygdalina* significantly enhanced fermentation performance in red sorghum beer. Increasing proportions of *V. amygdalina* resulted in higher apparent attenuation and ethanol production, with alcohol content increasing from 5.2% (v/v) in the hop-only control to 6.3% (v/v) in the 100% *V. amygdalina* formulation. Similar constraints in fermentability and alcohol yield have been widely reported for sorghum-based beers due to limited free amino nitrogen and starch accessibility (Taylor et al., 2006; Rouse et al0.2007).

The observed increase in apparent attenuation (61.6%–75.5%) indicates more efficient sugar utilization by *Saccharomyces cerevisiae.* Comparable improvements in ethanol production in sorghum malt worts supplemented with plant-derived additives have been reported previously (Agu and Palmer, 1998; Akinpelu, 1998). Moreover, enhanced fermentation performance in the presence of *V. amygdalina extracts* has been documented in earlier studies on sorghum beer fermentation (Amisi et al., 2023), supporting the consistency of the present findings under controlled conditions.

The mechanisms underlying the positive effects of *V. amygdalina* on yeast performance are likely multifactorial. Leaves of *V. amygdalina* are known to contain appreciable levels of minerals (iron, calcium, magnesium), vitamins, amino acids, and reducing sugars, which may act as supplementary nutrients for yeast metabolism (Farombi and Owoeye, 2011; Erasto et al., 2007). Such nutrients can enhance yeast vitality, glycolytic activity, and ethanol biosynthesis, particularly in nutrient-limited sorghum worts (Ijeh and Ejike, 2011; Ojimelukwe and Amaechi, 2019). At the same time, *V. amygdalina* is rich in polyphenols and sesquiterpene lactones, compounds widely reported for their antimicrobial properties (Ijeh and Ejike, 2011). While high concentrations of phenolic compounds may inhibit yeast activity, moderate levels have been shown to exert neutral or even stimulatory effects by inducing adaptive stress responses in *S. cerevisiae* (Farombi and Owoeye, 2011; Moreno-Arribas and Polo, 2009). The absence of fermentation inhibition in the present study suggests that the applied doses of *V. amygdalina* were within a favorable range, where nutritional and adaptive effects outweighed any inhibitory action.

The enhanced fermentation performance observed with increasing levels of *Vernonia amygdalina* may also be linked to its nutritional contribution to the wort. As reported by Herkenhoff et al. (2023), probiotic and non-conventional microorganisms in sour beer fermentations can modulate yeast metabolic activity through nutrient availability and metabolic interactions. Similarly, the mineral content, amino acids, and bioactive compounds present in *V. amygdalina* likely provided supplementary nutrients that accelerated *Saccharomyces cerevisiae* metabolism in the present study.

Polyphenols are increasingly recognized as modulators of yeast physiology during fermentation. Praia et al. (2022) reported improved fermentation performance and yeast vitality in fruit-supplemented sour beers, attributing these effects to moderate polyphenol concentrations that enhanced yeast stress tolerance and metabolic efficiency. These findings support our hypothesis that polyphenolic compounds present in *Vernonia amygdalina* contributed positively to yeast vitality and fermentation efficiency when applied at controlled substitution levels.

A progressive decrease in pH was observed during fermentation in all treatments, with significantly lower final pH values in beers containing higher proportions of *V. amygdalina*. Final pH values around 4.2 are typical for sorghum beers and contribute positively to microbial stability and shelf life (Rouse et al., 2007; Bamforth and Cook, 2005). The enhanced acidification observed in *V. amygdalina*-rich formulations may be attributed to increased yeast metabolic activity and organic acid production, as well as to the presence of acidic phenolic compounds naturally occurring in the plant (Farombi and Owoeye, 2011). Similar pH-lowering effects associated with plant-derived polyphenols have been reported in other fermented beverages (Rodríguez et al., 2009).

Beer color increased progressively with increasing substitution levels of *V. amygdalina*, reflecting the contribution of plant-derived pigments and polyphenolic compounds. Polyphenols are known to influence beer color through oxidation reactions and interactions with proteins and carbohydrates (Bamforth and Cook, 2005).

In conventional brewing, variations in hop cultivar, geographical origin, and processing conditions are recognized as major determinants of bitterness quality, polyphenolic composition, aroma profile, and technological behavior of beer, including fermentation performance (Van Holle et al., 2021; Herkenhoff et al., 2024a). Recent work by Herkenhoff et al. (2024a) showed that hops produced in different growing regions display distinct volatile fingerprints, resulting in measurable differences in beer aroma and fermentation dynamics. These observations provide a relevant framework for interpreting the present results, in which substitution of hops with *Vernonia amygdalina* altered fermentation kinetics. Rather than constituting a direct chemical replacement of hop-derived iso-α-acids, the use of *V. amygdalina* can be viewed as a modification of the bittering and polyphenolic matrix of the wort, consistent with current discussions on ingredient origin, terroir, and diversification of bittering agents in brewing science (Jeantet et al., 2007; Van Holle et al., 2021; Herkenhoff et al., 2024a).

The timing of *V. amygdalina* addition was a critical parameter influencing bitterness expression and aroma preservation. In this study, the plant material was added at the end of the 60-min boiling step, immediately before cooling, to favor extraction of bitter compounds while limiting thermal degradation of aroma-active constituents. Previous studies have shown that late additions better preserve volatile compounds, whereas prolonged boiling leads to their loss through evaporation and heat-induced degradation, despite continued transformation of bitterness precursors (Praet et al., 2015; Ocvirk and Košir, 2020). Limiting exposure of *V. amygdalina* compounds to extended high-temperature treatment likely reduced the risk of excessive vegetal or astringent character and contributed to the stable fermentation performance observed across substitution levels.

Microbiological quality control analyses indicated the absence of detectable contaminating microorganisms at the end of fermentation, while alcoholic fermentation proceeded normally. This selective behavior is functionally comparable to that of hop-derived iso-α-acids, which inhibit a broad range of Gram-positive bacteria without suppressing yeast activity (Simpson and Smith, 1992; Bamforth and Cook, 2005; Akinpelu, 1998; Jeantet et al., 2007). Previous studies have demonstrated antimicrobial activity of *V. amygdalina* extracts against lactic acid bacteria, coliforms, and spoilage microorganisms (Akinpelu, 1998; Ijeh and Ejike, 2011). This selective antimicrobial effect is consistent with previous reports demonstrating that *V. amygdalina* extracts inhibit a wide range of bacteria and fungi without negatively affecting brewer's yeast (Babalola and Okoh, 1996; Evbuomwan et al., 2018; Oboh and Masodje, 2009). The present results suggest that *V. amygdalina* may contribute to microbiological stability in sorghum beer brewing, although these findings should be interpreted as quality control outcomes rather than evidence of complete sterility.

The use of *V. amygdalina* as a hop substitute offers technological advantages for sorghum beer brewing, particularly in regions where hops are not locally available. Similar strategies aimed at replacing imported brewing inputs with indigenous plant resources have been proposed to enhance sustainability and economic resilience in African brewing systems (Taylor et al., 2006; Rouse et al., 2007).

Nevertheless, important limitations remain. Sensory properties of bitterness imparted by *V. amygdalina* were not evaluated, and the lack of phytochemical characterization limits mechanistic interpretation. Furthermore, laboratory-scale fermentations may not fully reflect industrial brewing conditions. Future work should integrate sensory analysis, detailed chemical profiling, and pilot-scale trials to determine optimal substitution levels and consumer acceptability.

## 4 Conclusion

This study demonstrates that *Vernonia amygdalina* can be effectively used as a partial or total substitute for hops in sorghum-based beer brewing, with significant effects on fermentation performance and key physicochemical properties. Increasing proportions of *V. amygdalina* enhanced alcohol production, apparent attenuation, acidity, and color intensity, indicating a stimulatory interaction between its phytochemical composition and *Saccharomyces cerevisiae* metabolism under the conditions tested.

The timing of addition was critical: *V. amygdalina* was added at the end of the 60-min boil, immediately before cooling, to maximize extraction of bitter compounds while preserving heat-sensitive aromatic molecules. This late addition likely contributed to balanced bitterness, preserved aroma, and consistent fermentation performance across substitution levels.

The results suggest that *V. amygdalina* functions not only as a bittering agent but also as a complex botanical ingredient that modulates the fermentation environment, similar to changes in hop variety or addition timing in conventional brewing. Its selective antimicrobial properties supported microbiological stability without inhibiting yeast activity.

However, the present work has important limitations. Sensory evaluation was not performed, and the qualitative nature of bitterness imparted by *V. amygdalina* relative to hops remains unknown. In addition, the absence of detailed phytochemical characterization prevents direct attribution of fermentation effects to specific compounds. Furthermore, the laboratory-scale fermentation conditions employed may not fully represent industrial brewing systems.

From a technological perspective, the present study demonstrates the feasibility of substituting hops with *Vernonia amygdalina* in sorghum beer fermentation. The work was intentionally designed to prioritize fermentation performance, physicochemical stability, and microbiological safety as a first validation step. Sensory evaluation and detailed phytochemical characterization were therefore not included at this stage, but are planned as a subsequent phase of research. Future studies will focus on sensory assessment, comprehensive profiling of bitter and polyphenolic compounds, and pilot- or industrial-scale trials to establish optimal substitution levels that balance fermentation efficiency, product quality, and consumer acceptability. This phased research strategy ensures that future sensory and chemical investigations are conducted on a robust, technologically validated fermentation basis.

## Ethics approval and consent to participate

Authors of this research did not involve human or animal subjects. So, no ethical approval is required.

Authors declare that this study has not required a consent to participate because it does not involve human subjects.

## Consent for publication

Authors declare that the manuscript does not contain any individual person's data in any form (including any individual details, images or videos). No consent to publish is required.

## CRediT authorship contribution statement

Arthur K. Amisi contributed to conception and design, acquisition of data, Analysis and interpretation of data, and in drafting the article. Material preparation and data collection were carried out by Nathan Luyeye and Exaucé Matundu under the supervision of Jean-Claude T. Bwanganga, Laboratory Manager. Guelor Kasereka participated in the analysis of the data collected. Jean-Paul Koto-Te-Nyiwa Ngbolua contributed in reviewing critically for significant intellectual content and the improvement of the draft written by Arthur Kapepa Amisi and all authors commented on previous versions of the manuscript. All authors have read and approved the final manuscript. Jean-Claude T. Bwanganga has supervised the study and gave the final approval of the version to be submitted and any revised version.

## Funding

The authors declare no funds, grants, or other support were received during the preparation of this manuscript.

## Declaration of competing interest

The authors have no relevant financial or non-financial interests to disclose.

They also declare that they have no conflict of interest.

The authors declare that we have no known competing financial interests or personal relationships that could have appeared to influence the work reported in this paper.

## Data Availability

The datasets generated and analyzed during the current study are available in the Zenodo repository (https://doi.org/10.5281/zenodo.18285308).
