# Supplementary material for: Vernonia amygdalina as a hop substitute in red sorghum beer: Effects on fermentation performance and physicochemical properties
Source: Curr Res Food Sci. 2026 Feb 2;12:101331. doi: 10.1016/j.crfs.2026.101331 (PMC12891878; doi:10.1016/j.crfs.2026.101331)
Supplement: Multimedia component 1 [file mmc1.docx]

**Supplementary Materials**

Supplementary tableau 1. Treatment summary

| Treatments | Hops (g) | *Vernonia amygdalina* (g) | wort (L) | Densité (°P) | Ratio Hops/V. amygdalina |
| --- | --- | --- | --- | --- | --- |
| E1 | 7 | 0 | 2 | 15 | 100 / 0 |
| E2 | 5.25 | 1.75 | 2 | 15 | 75/25 |
| E3 | 3.5 | 3.5 | 2 | 15 | 50/50 |
| E4 | 1.75 | 5.25 | 2 | 15 | 25/75 |
| E5 | 0 | 7 | 2 | 15 | 0/100 |

Supplementary table 2. Analysis of variance of pH versus time (h) and seeding dose

| Source | DF | SS | MS | F | P |
| --- | --- | --- | --- | --- | --- |
| Time (h) | 8 | 21.2611 | 2.6576 | 156.74 | 0.000 |
| Dose (%) | 4 | 0.7985 | 0.1996 | 11.77 | 0.000 |
| Error | 122 | 2.0685 | 0.0170 |  |  |
| Total | 134 | 24.1282 |  |  |  |

S = 0.130212 R-Sq = 91.43% R-Sq(adj) = 90.58%

This analysis shows that the inoculation dose and fermentation time have a highly significant effect on pH, as shown by their respective p-values, all of which are less than 0.05.

Supplementary table 3. Analysis of variance of Brix degree versus time (h) and seeding dose

| Source | DF | SS | MS | F | P |
| --- | --- | --- | --- | --- | --- |
| Time (h) | 8 | 1063.941 | 132.993 | 470.53 | 0.000 |
| Dose (%) | 4 | 15.007 | 3.752 | 13.27 | 0.000 |
| Error | 122 | 34.483 | 0.283 |  |  |
| Total | 134 | 1113.431 |  |  |  |

S = 0.531642 R-Sq = 96.90% R-Sq(adj) = 96.60%

The results indicate that fermentation time strongly influences the Brix percentage, as does the inoculation dose. These two factors have significant effects with p-values well below the 5% threshold.

Supplementary table 4. Analysis of variance of staining (EBC) vs. time (h) and seeding dose

| Source | DF | SS | MS | F | P |
| --- | --- | --- | --- | --- | --- |
| Time (h) | 8 | 46.1598 | 5.7700 | 1472.84 | 0.000 |
| Dose (%) | 4 | 61.3882 | 15.3470 | 3917.49 | 0.000 |
| Error | 122 | 0.4779 | 0.0039 |  |  |
| Total | 134 | 108.0259 |  |  |  |

The analysis reveals that coloring is strongly influenced by duration and dosage, with extremely low p-values associated with these two factors, confirming their significant effect.

Supplementary table 5. Analysis of density (°P) versus dtime (h) and seeding dose

| Source | DF | SS | MS | F | P |
| --- | --- | --- | --- | --- | --- |
| Time (h) | 8 | 1494.56 | 186.82 | 361.86 | 0.000 |
| Dose (%) | 4 | 76.58 | 19.14 | 37.08 | 0.000 |
| Error | 122 | 62.99 | 0.52 |  |  |
| Total | 134 | 1634.13 |  |  |  |

S = 0.718527 R-Sq = 96.15% R-Sq (adj) = 95.77%

Density is also significantly affected by fermentation duration and inoculation dose, with p-values below 0.05 for both factors.

Supplementary table 6. Analysis of actual extract versus time (h) and seeding dose

| Source | DF | SS | MS | F | P |
| --- | --- | --- | --- | --- | --- |
| Time (h) | 8 | 1004.452 | 125.557 | 361.86 | 0.000 |
| Dose (%) | 4 | 51.467 | 12.867 | 37.08 | 0.000 |
| Error | 122 | 42.331 | 0.347 |  |  |
| Total | 134 | 1098.250 |  |  |  |

S = 0.589048 R-Sq = 96.15% R-Sq(adj) = 95.77%

Time and dose have a highly significant effect on the actual extract. This conclusion is based on very low p-values for both parameters studied.

Supplementary table 7. Analysis of attenuation versus time (h) and inoculation dose

| Source | DF | SS | MS | F | P |
| --- | --- | --- | --- | --- | --- |
| Time (h) | 8 | 687167 | 85896 | 114.94 | 0.000 |
| Dose (%) | 4 | 40890 | 10222 | 13.68 | 0.000 |
| Error | 122 | 91176 | 747 |  |  |
| Total | 134 | 819232 |  |  |  |

S = 27.3375 R-Sq = 88.87% R-Sq(adj) = 87.78%

The analysis of variance reveals that attenuation is significantly influenced by fermentation duration and inoculation dose, with both p-values well below 0.05, or 5%.

*Supplementary table 8. Analysis of alcohol content (v/v) versus time (h) and inoculation dose*

| Source | DF | SS | MS | F | P |
| --- | --- | --- | --- | --- | --- |
| Time (h) | 8 | 467.520 | 58.440 | 399.19 | 0.000 |
| Dose (%) | 4 | 14.248 | 3.562 | 24.33 | 0.000 |
| Error | 122 | 17.860 | 0.146 |  |  |
| Total | 134 | 499.628 |  |  |  |

S = 0.382616 R-Sq = 96.43% R-Sq(adj) = 96.07%


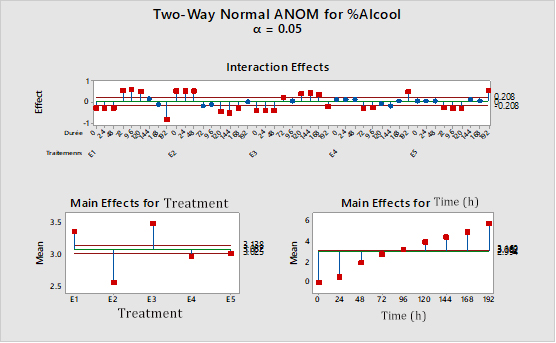


(a)

(c)

(b)

*Supplementary figure 1. (a): Interaction treatment vs time (h), (b) Average alcohol content percentage for the treatments, (c): evolution of alcohol content over time (h)*

Supplementary Figure 1a (top graph) shows the interactions between the Treatments and time factors on the percentage of alcohol. The red dots outside the decision lines (±0.208) indicate significant interactions. If most of the dots are close to zero and within the limits, this means that the interaction between the factors is not significant.

Supplementary Figure 1b (bottom left graph) shows the average alcohol percentage for the different treatments (E1 to E5). The red horizontal lines represent the overall average. If a treatment is outside the decision lines, it means that it has a significant effect. Here, E3 appears to be significantly lower, while E2 is higher than average.

Supplementary Figure 1c (bottom right graph) shows the evolution of the alcohol percentage as a function of fermentation time. A gradual increase can be observed, with a plateau after a certain amount of time. A longer fermentation time tends to increase the alcohol percentage, but after 192 hours, the effect appears to stabilize.
